# Exploring the Characteristics of Circulating Tumor DNA in Pt1a Clear Cell Renal Cell Carcinoma: A Pilot Study

**DOI:** 10.3390/cancers15133306

**Published:** 2023-06-23

**Authors:** Hongkyung Kim, Jee Soo Park, Zisun Choi, Seungki Min, Jihyang Park, Saeam Shin, Jong Rak Choi, Seung-Tae Lee, Won Sik Ham

**Affiliations:** 1 Department of Laboratory Medicine, Chung-Ang University Gwangmyung Hospital, Chung-Ang University College of Medicine, Gwangmyung 14353, Republic of Korea; khk.labmed@cauhs.or.kr; 2 Department of Urology and Urological Science Institute, Yonsei University College of Medicine, Severance Hospital, Seoul 03722, Republic of Korea; jsparkysmed@gmail.com; 3 Department of Urology, Sorokdo National Hospital, Goheung 59562, Republic of Korea; 4 Dxome, Seongnam 13558, Republic of Korea; zschoi@dxome.com (Z.C.); skmin@dxome.com (S.M.); jhpark@dxome.com (J.P.); cjr0606@yuhs.ac (J.R.C.); 5 Department of Laboratory Medicine, Yonsei University College of Medicine, Severance Hospital, Seoul 03722, Republic of Korea; saeam0304@yuhs.ac

**Keywords:** circulating tumor DNA, clear cell renal cell carcinoma, pT1a, small renal mass, next-generation sequencing

## Abstract

**Simple Summary:**

Circulating tumor DNA (ctDNA) has emerged as a potential biomarker for clear cell renal cell carcinoma (ccRCC). However, the ctDNA characteristics have not been demonstrated in small ccRCC. The aim of our pilot study is to explore the characteristics of ctDNA in pT1a ccRCC (ccRCC less than 4 cm in diameter). We included 53 patients with pT1a ccRCC, a greater number than in previous studies using next-generation sequencing. We found that the ctDNA detection rate was low in pT1a ccRCC. The relationship between ctDNA and clinicopathological features, such as tumor size, tumor grade, and patient age, was not clear. Increasing the sensitivity and removing background noise in ctDNA analysis may aid in further understanding the characteristics of ctDNA, thereby enhancing its clinical utility in pT1a ccRCC.

**Abstract:**

Circulating tumor DNA (ctDNA) is a promising biomarker for clear cell renal cell carcinoma (ccRCC); however, its characteristics in small renal masses of ccRCC remain unclear. In this pilot study, we explored the characteristics of ctDNA in pT1a ccRCC. Plasma samples were collected preoperatively from 53 patients with pT1a ccRCC. The ctDNA of pT1a ccRCC was profiled using next-generation sequencing and compared with that of higher-stage ccRCC. The association of ctDNA in pT1a ccRCC with clinicopathological features was investigated. The positive relationship of mutations between ctDNA and matched tissues was evaluated. In pT1a ccRCC, the ctDNA detection rate, cell-free DNA concentration, and median variant allele frequency were 20.8%, 5.8 ng/mL, and 0.38%, respectively, which were significantly lower than those in metastatic ccRCC. The ctDNA gene proportions in pT1a samples differed from those in metastatic ccRCC samples. The relationships between ctDNA and tumor size, tumor grade, and patient age were not elucidated. The positive concordance between ctDNA and matched tissues was poor for pT1a ccRCC. Strategies are needed to increase sensitivity while eliminating noise caused by clonal hematopoiesis to increase the clinical utility of ctDNA analysis in small renal masses of ccRCC.

## 1. Introduction

Renal cell carcinoma (RCC) is a type of cancer originating from renal tubular epithelial cells. Clear cell renal cell carcinoma (ccRCC) is the most common subtype of RCC and the main cause of death attributed to kidney cancer [1]. Over the past two decades, the global incidence of RCC has increased by 2% annually. The number of new kidney cancer cases and deaths in the United States in 2022 was expected to be 79,000 and 13,920, respectively [2,3]. Furthermore, nephrectomies are mainly performed for localized ccRCC; however, 30% of patients eventually develop metastasis after surgery [1,4]. Additionally, the 5-year survival rate was found to be 95%, 88%, and 59% for stages 1, 2, and 3, respectively, which decreases to 20% in patients with distant metastases [1].

As sonography and computed tomography (CT) have become widely used, incidental findings of small renal masses (SRMs; ≤4 cm in diameter) have increased, accounting for more than half of the newly diagnosed cases [5]. Approximately 20% of SRMs are benign, while 60% are malignant and indolent with low metastatic potential [6,7]. The remaining 20% of SRMs are malignant with unfavorable characteristics [8,9]. Therefore, risk stratification at diagnosis is necessary for the proper management of SRMs, particularly for active surveillance and ablative therapies [1,6].

A biopsy is essential for the diagnosis of SRMs and can provide important information, such as tumor grade and necrosis status, for clinical decision-making [4]. However, several limitations of biopsy should be addressed. First, up to 14% of renal mass biopsies are non-diagnostic [8,9]. Second, a renal mass biopsy may not fully characterize the entire renal mass owing to intratumoral heterogeneity [8,10]. Third, although renal mass biopsies show an acceptable concordance rate for histology in surgical specimens, the concordance of grades is less reliable [8,9]. Finally, safety and tumor seeding issues remain associated with renal mass biopsies, especially when biopsies are performed for renal masses with cystic changes [8]. Therefore, several studies have been conducted to find alternative or complementary methods to evaluate conventional clinicopathological parameters and ultimately discover new biomarkers for the diagnosis and risk stratification of SRMs [11,12].

Circulating tumor DNA (ctDNA) is found in the bloodstream and refers to DNA derived from tumor cells. As ctDNA contains information on genetic modifications in cancer cells, it is a potential biomarker for several cancers with various applications in the diagnosis, monitoring, treatment, and prognosis of cancer [13]. Moreover, this non-invasive method can capture the entire genetic heterogeneity and burden of cancer [14]. Many studies have been conducted on the clinical application of promising ctDNA analysis in RCC, the majority of which have focused on ccRCC. The ctDNA mutational profile of ccRCC and its prognostic value have been discussed. However, most ctDNA studies have been conducted on metastatic (M1) ccRCC, and little information is available on the characteristics of ctDNA in localized small ccRCC [10,15,16,17]. In this pilot study, we explored the characteristics of ctDNA in ccRCC-SRMs (pT1a).

## 2. Materials and Methods

### 2.1. Samples and Study Design

The study protocol was approved by the Institutional Review Board/Ethics Committee of Yonsei University College of Medicine, Seoul, Republic of Korea (approval no: 4-2019-1039), and the requirement for informed consent was waived by the Institutional Review Board/Ethics Committee of Yonsei University College of Medicine due to the retrospective study design. All methods were performed in accordance with the relevant guidelines and regulations.

A total of 53 patients with pT1a ccRCC were enrolled in this study. Mutational profiles of ctDNA in pT1a ccRCC and relationships between ctDNA detection and clinicopathological features (tumor size, tumor grade, and age) were investigated. To compare their ctDNA features (detection rate, cell-free DNA [cfDNA] concentration, variant allele frequency [VAF], and proportion of specific genes with ctDNA) with those of patients with higher stages, five patients with pT1b, three with pT2a, six with pT3a, and eight with M1 ccRCC were enrolled, and 10 patients with benign renal tumors were included as controls. Peripheral blood was collected immediately before surgical resection. The blood samples were aliquoted into ethylenediaminetetraacetic acid-containing tubes, centrifuged at 1600× *g* for 10 min at 4 °C, and transferred to fresh tubes. Then, the samples were further centrifuged at 4000× *g* for 10 min at 4 °C, and the plasma samples were stored at −80 °C until ctDNA analysis. Tumor tissues were obtained from eight patients (four with pT1a, one with pT3a, two with M1, and one with a benign renal tumor) to compare the concordance of somatic mutations between the plasma and tissues. All samples in this study were obtained between February 2020 and December 2020.

The data on age, sex, pathological data, and imaging studies were obtained via medical record investigation. Histological subtypes were assessed according to the 2016 edition of the World Health Organization (WHO) histological classification of renal tumors. Tumor grade was determined according to the Furhman or WHO/International Society of Urological Pathology grading systems. Finally, the stage was assessed based on the 8th edition of the American Joint Committee on Cancer Staging Manual.

### 2.2. Library Preparation, Target Capture, and Sequencing for Plasma Samples

Circulating cfDNA was extracted from 3–4 mL of plasma samples using a Magnetic Serum/Plasma Circulating DNA Kit (Dxome, Seongnam, Republic of Korea). The size of the cfDNA was measured using a TapeStation 4200 (Agilent Technologies, Santa Clara, CA, USA). The concentration of the cfDNA was measured using a Qubit 3.0 Fluorometer (Thermo Fisher Scientific, Waltham, MA, USA). For library preparation, 5–30 ng of cfDNA was used, except for one sample of 4.5 ng. Library preparation was conducted using the DxSeq Library prep reagent (Dxome). Sequencing libraries were hybridized with customized probes targeting 16 RCC-related genes, which are frequently mutated in RCC, as shown in previous studies (Supplementary Table S1) [18,19]. The enriched DNA was amplified, and clusters were generated and sequenced on a NovaSeq 6000 System (Illumina, San Diego, CA, USA) with 2 × 151 bp reads targeting 35,000× average sequencing depth per sample. All procedures were performed according to the manufacturer’s instructions.

The position index sequencing (PiSeq, Dxome) algorithm was used to call and annotate ctDNA mutations. PiSeq was designed to accurately detect mutations with a low VAF using the positional information of the sequencing reads [20]. The VAF was calculated as (read depth count of identified variant/total read depth count at the position) × 100. All mutations were manually inspected using an Integrative Genomic Viewer [21].

### 2.3. Tissue Sequencing

Of the eight tumor tissues, five were frozen tissue samples and three were formalin-fixed paraffin-embedded (FFPE) tissues. DNA was extracted from the frozen tissue samples using the QIAamp DNA Blood Mini Kit (Qiagen, Hilden, Germany) and from the FFPE tissue using the QIAGEN AllPrep FFPE Kit (Qiagen). DNA from the frozen tissues was sequenced using the Twist Human Core Exome Kit (Twist Bioscience, San Francisco, CA, USA), and DNA from the FFPE tissues was sequenced using the TruSight Oncology 500 (Illumina). After hybridization, paired-end sequencing with 2 × 151 bp reads was performed using a NovaSeq 6000 System (Illumina) for DNA from both types of tissues. All procedures were performed according to the manufacturer’s instructions.

### 2.4. Statistical Analysis

Fisher’s exact test was performed to compare the detection rates between the pT1a and higher-stage groups and to compare the proportion of high-grade tumors according to ctDNA detection in pT1a ccRCC. The Wilcoxon rank-sum test was performed to compare the tumor size, patient age, cfDNA concentration, and VAF among the groups. All statistical analyses were conducted using R (version 4.2.1; R Core Team, 2022), and a *p*-value < 0.05 was considered statistically significant.

## 3. Results

### 3.1. Patient Characteristics

The clinicopathological information of the 53 patients with pT1a ccRCC is shown in Table 1. The cohort included 37 (69.8%) males and 16 (30.2%) females, and 20 (37.7%) patients were over 60 years of age. The median size of the pT1a ccRCC tumors was 1.9 cm (interquartile range, 1.3–2.8). Additionally, 20 (37.7%) of the ccRCC cases presented with grade 3/4 tumors. Information on the patients with M1, pT1b-3a ccRCC, and benign renal tumors is shown in Supplementary Table S3, Supplementary Table S4, and Supplementary Table S5, respectively.

### 3.2. Mutations in ctDNA

**Figure 1:**
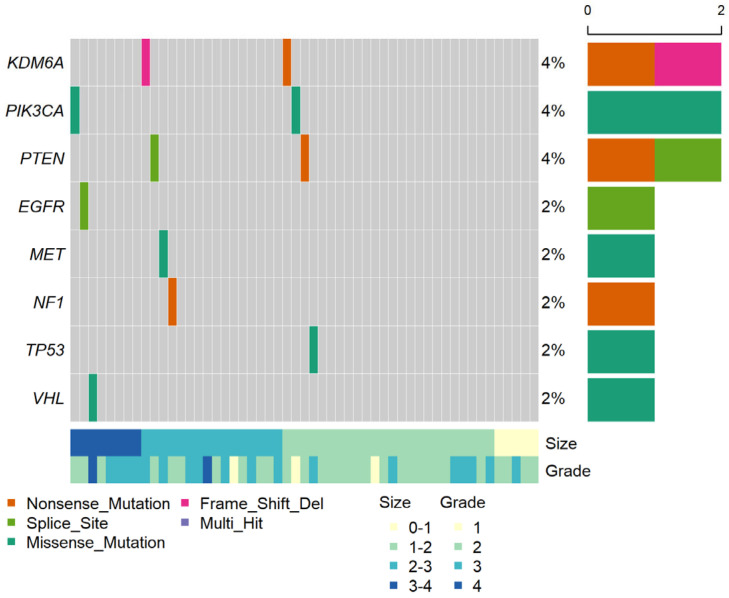
Genomic profile of ctDNA in 53 patients with pT1a ccRCC. The middle heat map shows ctDNA detection events, with their percentages among the total number of patients on the right. The top histogram shows the number of ctDNA variants per sample; the right histogram shows the number of ctDNAs per gene; the bottom heat map shows information about the tumor size and tumor grade per sample.

The sequencing for pT1a yielded a median on-target read of 59.2% (58.3–60.8) and an average depth of 19,946 × (15,694–31,820) (Supplementary Table S2). Genetic alterations in the ctDNA were profiled using PiSeq for those with pT1a ccRCC (Figure 1). The detailed ctDNA information and patient characteristics are shown in Supplementary Table S6. Specifically, ctDNA was detected in 11 of the 53 patients with pT1a ccRCC (detection rate, 20.8%). All patients with ctDNA had a single ctDNA. Two ctDNAs were found in *KDM5A*, *PIK3CA,* and *PTEN*, respectively, while one ctDNA was found in *MET*, *NF1*, *TP53,* and *VHL*, respectively. The minimum and maximum VAFs were 0.115% for *PIK3CA* and 2.749% for *PTEN*. The median VAF of the pT1a ccRCC samples was 0.378% (0.258–0.488%). The ctDNA information for M1 and pT1b-3a ccRCC is provided in Supplementary Tables S3 and S4, respectively. Among the 10 patients with benign tumors, one patient with a large pseudocyst was found to have a *MET* mutation (Supplementary Table S5).

### 3.3. Relationship between ctDNA and Clinicopathological Features

**Figure 2:**
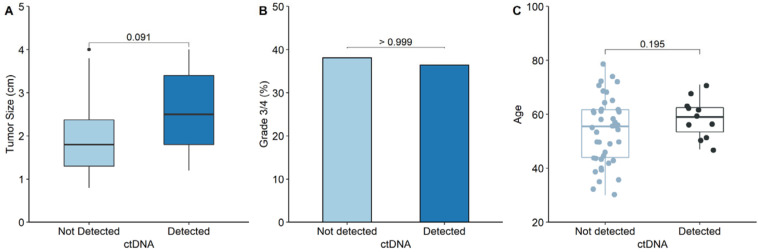
Comparison of the clinicopathological features between groups with and without ctDNA detection in pT1a ccRCC. (**A**) Comparison of the median primary tumor size between ctDNA detection and non-detection groups. (**B**) Comparison of the proportion of grade 3/4 between ctDNA detection and non-detection groups. (**C**) Comparison of patient’s age between ctDNA detection and non-detection groups.

Among those with pT1a ccRCC, the tumor size was compared between the ctDNA detection and non-detection groups (Figure 2A). The median tumor size in the ctDNA detection group was larger than that in the non-detection group (2.5 cm vs. 1.8 cm, respectively; *p* = 0.091), but the difference was not statistically significant. Additionally, the proportion of tumor grade 3/4 among those with pT1a ccRCC was compared between the ctDNA and non-ctDNA detection groups (Figure 2B). As a result, ctDNA was detected in 4 of the 20 patients (20.0%) with tumor grade 3/4, and in 7 of 33 patients (21.2%) with tumor grade 1/2. No relationship was observed between ctDNA detection and tumor grade (*p* > 0.999). Figure 2C shows a comparison of patient age between the ctDNA detection and non-detection groups among those with pT1a ccRCC. The median ages of the two groups were not significantly different (59.0 years vs. 55.5 years, *p* = 0.195).

### 3.4. Comparison of ctDNA Characteristics between pT1 and M1 ccRCC

**Figure 3:**
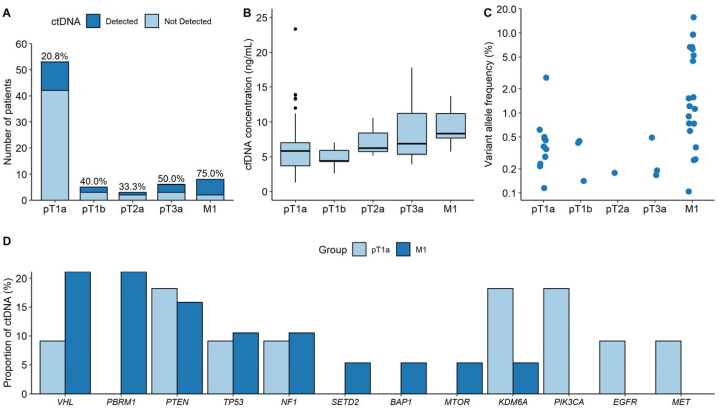
Comparison of the ctDNA characteristics between pT1a and higher-stage ccRCC. (**A**) Detection rates. (**B**) cfDNA concentrations. (**C**) Variant allele frequencies. (**D**) Proportions of genes with ctDNA between pT1a and M1 ccRCC groups.

Figure 3A shows the detection rate of pT1a compared with that of higher stages. The detection rate generally increased from pT1a to M1 ccRCC, with the exception of pT2a. Additionally, the detection rate of pT1a was significantly lower than that of M1 ccRCC (20.8% vs. 75.0%; *p* = 0.0043). Figure 3B shows cfDNA concentrations according to the stage. The median cfDNA concentration of pT1a ccRCC was significantly lower than that of M1 (5.8 ng/mL vs. 8.3 ng/mL, *p* = 0.008). Figure 3C demonstrates that the VAFs in the pT1a samples were significantly lower than those in the M1 samples (0.378% vs. 1.210%, *p* = 0.006). Figure 3D shows the proportion of genes with ctDNA in pT1a samples compared with that in M1 ccRCC samples. The proportion of specific genes with ctDNA was calculated by dividing the number of ctDNAs detected by a specific gene by the total number of detected ctDNA mutations. In contrast to pT1a ccRCC, *VHL,* and *PBRM1* were the most frequently detected genes in patients with M1 ccRCC (21.1% [4/19]).

### 3.5. Positive Concordance of Mutations between ctDNA and Matched Tissue

**Figure 4:**
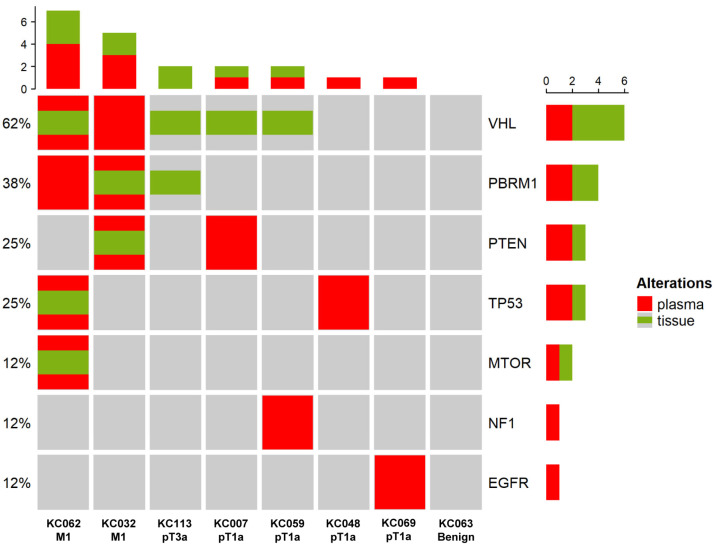
Positive concordance of mutations between ctDNA and matched tissue. Information on the stages is shown at the bottom of the heatmap. Red refers to mutations detected in the plasma, while green refers to mutations detected in the tissue. The histogram at the top shows the number of mutations detected in each patient. The histogram on the right shows the number of mutations detected per gene, with each ratio indicated on the left of the heatmap.

The positive concordance of mutations between ctDNA and matched tissues in ccRCC is shown in Figure 4. Of the eight patients, four had pT1a ccRCC, two had M1 ccRCC, one had pT3a ccRCC, and one had a benign renal tumor. Positive concordant mutations were identified in both patients with M1 ccRCC. No concordant mutations were observed in the patients with pT1a or pT3a ccRCC. One patient with M1 ccRCC did not exhibit a mutation of *PBRM1* in the tissue, but ctDNA of *PBRM1* was observed in the plasma. Similarly, the other patient with M1 ccRCC had a *VHL* mutation in the plasma that was not observed in the tissue. *VHL* mutations were detected in the tissues of two patients with pT1a; however, no concordant mutations were detected in the plasma. Finally, *VHL* and *PBRM1* mutations were detected in the tissue of a patient with pT3a ccRCC, but no concordant mutations were detected in the plasma.

## 4. Discussion

In several ctDNA studies on RCC, mutational profiles and their concordance with matched tissues have been reported. The role of ctDNA in predicting prognosis, treatment response, and resistance to RCC has also been investigated. However, most of these studies were conducted on metastatic or large-sized ccRCC [17]. Table 2 indicates that the present study included a greater number of patients with localized small ccRCC than previous studies using next-generation sequencing technology [10,15,16].

In the present study, we used a sensitive RCC-specific next-generation sequencing (NGS) panel that enabled the detection of mutations with low VAF (0.25%). The ctDNA detection rate in pT1a ccRCC was 20.8%, which was significantly lower than that in M1 ccRCC. This result was reasonable, given that ctDNA levels may suggest more advanced disease and a higher tumor mutational burden [22]. Moreover, because the amount of ctDNA in plasma correlates with tumor size, the relatively small size of tumors in the present study may contribute to the low ctDNA detection rate. Notably, the percent of ctDNA can be lower than 0.1% in the peripheral blood when the tumor size is less than 2.4 cm in diameter [23]. Additionally, the type of cancer significantly affects the amount of ctDNA in the plasma. The amount of ctDNA released into the bloodstream is determined by the characteristics of the cancer, such as tumor vascularization and histological type. RCC has been classified as a low-ctDNA cancer by several studies, but the cause of low ctDNA levels has not been clearly established [10,24,25].

Tumor size is an important prognostic factor for RCC [1]. Although the median tumor size was larger when ctDNA was detected in pT1a ccRCC, this difference was not statistically significant. Tumor grade is another critical factor in RCC that predicts metastasis and decreases overall survival [1,26]. However, no relationship was observed between ctDNA and tumor grade 3/4 in pT1a ccRCC. The low ctDNA detection rate may mask the potential relationship between ctDNA and these pathologically important features.

The proportion of genes with ctDNA in pT1a ccRCC differed from that in M1 ccRCC. The most frequently mutated gene in ccRCC is *VHL*, followed by *PBRM1*, *SETD2*, *BAP1*, and *KDM5C* [19,27,28]. Although the proportion of genes in M1 ccRCC correlated with well-established mutational profiles, only one mutation was detected in *VHL* in pT1a ccRCC. Because *VHL* is considered an early evolutionary ancestor gene of ccRCC, it was unexpected that *VHL* mutations were less frequently detected in pT1a [27].

*EGFR, NF1, PIK3CA,* and *TP53,* whose mutations have been detected in pT1a cancer, are common driver genes in many cancers [29]. Among them, *TP53* mutations are recognized as one of the drivers of ccRCC and have also been observed in subclonal events of ccRCC [27]. A genomic meta-analysis of ccRCC demonstrated that *TP53* mutations were more prevalent at metastatic sites [28]. Additionally, several ctDNA studies have shown that *TP53* is one of the most frequent ctDNA-related genes in metastatic RCC [30]. Therefore, the ctDNA of *TP53* may be a potential biomarker for predicting poor prognosis in ccRCC-SRMs. A long-term prospective study is required to determine the clinical significance of ctDNA in ccRCC-SRMs. Because the 5-year cancer-specific and 5-year recurrence-free survival rates in patients with pT1a RCC were 97% and 96%, respectively, the clinical outcome could not be evaluated in the current study because of the short follow-up period (<2 years after surgery) [31].

In the current study, the possibility that ctDNA mutations were clonal hematopoiesis of indeterminate potential (CHIP) could not be ruled out. Approximately 9–31% of somatic mutations in ccRCC have been identified as CHIP [29,32]. Although CHIP mutations can act as background noise in ctDNA analysis, matched peripheral blood mononuclear cells were not sequenced to confirm CHIP, and major CHIP genes such as *DNMT3A*, *TET2*, and *ASXL1* were not included in the targeted NGS panel in the present study [14,33]. Possible CHIP mutations may affect the detection rates and complicate the interpretation of ctDNA analysis. Although there were no concordant mutations in matched tissues for all plasma-detected mutations in pT1a ccRCC, the intratumoral heterogeneity of ccRCC may lead to discrepancies in mutation detection between plasma and matched tissues [8,10]. In addition, CHIP generally increases with age, and approximately 6% of individuals in their 60s were found to have clonal somatic mutations [33]. Although the analysis of the association between age and ctDNA detection alone was insufficient to determine the presence of CHIP mutations, there was no significant difference in age between the ctDNA detection and non-detection groups.

Several concerns should be addressed regarding the feasibility of ctDNA analysis of ccRCC-SRMs. First, poor reproducibility was expected. High reproducibility of ctDNA analysis is typically guaranteed when mutations with a VAF > 0.5% are detected [34]. Unlike in M1 ccRCC, the VAFs of most ctDNA mutations were <0.5% in pT1a cases. Second, the positive concordance rate of mutations between plasma and matched tissues was poor in pT1a ccRCC. Although only four matched tissue samples were used to identify mutations concordant with ctDNA in pT1a ccRCC, two matched tissue samples showed *VHL* mutations, which are the most frequently mutated genes in ccRCC, with the potential to be detected in plasma [19]. A previous study that included 10 localized ccRCC samples with tumor sizes greater than those in the present study showed similar outcomes between ctDNA and matched tissue. In this previous study, *VHL* mutations were detected in more than half of the matched tissue samples; however, only one-third of concordant *VHL* mutations were detected in the plasma [16]. Third, ctDNA was detected in one patient with a benign tumor. Thus, potential false positives should be interpreted with caution during ctDNA analyses. Although ctDNA from benign tumors is unlikely to be detected by ctDNA analysis, cfDNA can be released in patients with benign or inflammatory diseases, resulting in overdiagnosis [24,35]. As the sample size of the benign control group was too small to evaluate the specificity of ctDNA, further studies are needed to demonstrate the effects of benign renal tumors on ctDNA analysis.

This study had several limitations. First, the characteristics of ctDNA in pT1a ccRCC have not been completely elucidated. Although the sample size of pT1a ccRCC patients was greater than that previously reported in the literature, the number of ctDNAs detected was too small to fully reveal the characteristics of ctDNA. In the future, strategies should be applied to improve the sensitivity of ctDNA analysis, such as ultra-deep sequencing, size selection of cfDNA fragments, and combining other types of genetic alterations (e.g., copy number variation and methylation) [36]. The ctDNA analysis from urine samples can be used to detect ctDNA that could not be detected in blood samples [10]. Therefore, using both urine and blood samples for ctDNA analysis can also increase its sensitivity. In Addition, a comprehensive NGS panel is required for a deeper analysis of ctDNA molecular characteristics. Second, the sample size of higher-stage ccRCC was relatively small to compare the characteristics of ctDNA with those of pT1a ccRCC. Third, since biopsy was not always performed on multiple sites of a renal tumor, some mutations in the matched tissue may have been missed. Moreover, mutations in matched tissues were only compared in selected patients. These factors could have affected the concordance of mutations between plasma and matched tissue samples. Fourthly, the applicability of ctDNA analysis in this study is somewhat limited due to its sole focus on ccRCC as the histological subtype for SRMs. While ccRCC is the predominant subtype of RCC, the inclusion of non-ccRCC, such as papillary, chromophobe, and oncocytoma subtypes, in future research will provide a more comprehensive perspective, thus broadening the utility and the scope of ctDNA analysis in SRMs [1]. Lastly, ctDNA analysis was conducted only prior to surgery, without any follow-up ctDNA analysis post-operation. Detecting residual ctDNA after surgery could be beneficial for patient management [13]. Furthermore, serial ctDNA analysis may provide significant insights into disease progression [10,16]. Therefore, we propose that further investigations should incorporate follow-up ctDNA analysis post-surgery for ccRCC-SRMs to enhance the comprehensive understanding of the disease course.

## 5. Conclusions

We explored the characteristics of ctDNA in pT1a ccRCC. We found pT1a ccRCC to be characterized by a low detection rate, low cfDNA concentration, low VAF, and different proportions of genes with ctDNA compared with M1 ccRCC and previously reported mutational profiles. The relationship of ctDNA with tumor size and grade remains unclear. Prospective studies are required to demonstrate the clinical significance of ctDNA in ccRCC-SRMs. Finally, improving the sensitivity and filtering of CHIP mutations in ctDNA analysis may help further elucidate the characteristics of ctDNA, thereby enhancing the clinical utility of ctDNA analysis in ccRCC-SRMs.

## Figures and Tables

**Table 1:** Clinicopathological information of 53 patients with pathological T1a ccRCC.

Variable	*n* (%)
Sex	
Male	37 (69.8)
Female	16 (30.2)
Age	
<40	6 (11.3)
40–49	10 (18.9)
50–59	17 (32.1)
60–69	14 (26.4)
≥70	6 (11.3)
Tumor size	
<1 cm	5 (9.4)
1–2 cm	24 (45.3)
2–3 cm	16 (30.2)
3–4 cm	8 (15.1)
Tumor grade	
1	3 (5.7)
2	30 (56.6)
3	18 (33.9)
4	2 (3.8)
Total	53 (100)

**Table 2:** Comparison of ctDNA detection rates in localized and metastatic ccRCC.

Reference	Localized			Metastatic	
	Patient, *n*	Median Tumor Size (cm)	ctDNA Detection (%)	Patient, *n*	ctDNA Detection (%)
Yoshiyuki Yamamoto (2019) [15]	14	–	14.3	39	35.9
Christopher G. Smith (Cohort Diamond) (2020) [10]	24	6.1	48.3	5	80.0
Yeon Jeong Kim (2021) [16]	10	7.1	40.0	8	50.0
Present study	53	1.9	20.8	8	75.0

## Data Availability

The data underlying this study are available in this published article and its Supplementary Material file, and they are also available in Figshare with the identifier https://doi.org/10.6084/m9.figshare.22331314 (accessed on 22 June 2023).

## Supplementary Materials

The following supporting information can be downloaded at: https://www.mdpi.com/article/10.3390/cancers15133306/s1. Table S1: Gene list of the target panel for renal cell carcinoma; Table S2: Sequencing statistics for plasma samples; Table S3: Information of patients with metastatic ccRCC; Table S4: Information of patients with pT1b-3a ccRCC; Table S5: Information of patients with benign renal tumor; Table S6: List of ctDNA variants of patients with pT1a ccRCC.
