# Supplementary material for: Exploring the Characteristics of Circulating Tumor DNA in Pt1a Clear Cell Renal Cell Carcinoma: A Pilot Study
Source: Cancers (Basel). 2023 Jun 23;15(13):3306. doi: 10.3390/cancers15133306 (PMC10341247; doi:10.3390/cancers15133306)
Supplement: Supplementary file 1 [file cancers-15-03306-s001.zip › cancers-2446298-supplementary.pdf]

Supplementary Tables

Supplementary Table S1. Gene list of the target panel for renal cell carcinoma

ARID1A	*BAP1*	*EGFR*	*ELOC*
KDM5C	*KDM6A*	*MET*	*MTOR*
NF1	*NF2*	*PBRM1*	*PIK3CA*
PTEN	*SETD2*	*TP53*	*VHL*

Supplemental Table S2. Sequencing statistics for plasma samples

Stage		Duplicates (%)	Average depth	On target (%)	% Covered (>100×)	% Covered (>200×)	% Covered (>300×)	% Covered (>500×)	% Covered (>1000×)
pT1a (n = 53)	Median	83.6	19,946	59.2	100.0	99.9	99.7	97.6	72.6
	[1Q-3Q]	[79.0-90.1]	[15,694-31,820]	[58.3-60.8]	[99.9-100.0]	[99.7-99.9]	[98.9-99.8]	[87.5-99.5]	[2.1-86.6]
pT1b (n = 5)	Median	83.6	26,966	59.3	100.0	99.9	99.8	98.8	68.1
	[1Q-3Q]	[83.0-89.8]	[20,356-30,886]	[58.1-60.1]	[100.0-100.0]	[99.9-99.9]	[99.7-99.8]	[96.8-99.0]	[67.0-80.5]
pT2a (n = 3)	Median	85.2	22,399	58.2	100.0	99.9	99.9	99.8	95.9
	[1Q-3Q]	[82.1-88.0]	[20,993.5-45,204.5]	[57.0-60.9]	[100.0-100.0]	[99.9-100.0]	[99.8-99.9]	[98.4-99.8]	[81.9-97.2]
pT3a (n = 6)	Median	80.9	21,129.5	60.7	100.0	99.9	99.7	97.8	77.3
	[1Q-3Q]	[77.7-84.6]	[16,799.3-30,002.5]	[57.6-61.3]	[100.0-100.0]	[99.8-99.9]	[99.6-99.7]	[97.0-98.6]	[77.1-89.1]
M1 (n = 8)	Median	79.7	18,707.5	57.7	100.0	99.9	99.9	99.4	93.1
	[1Q-3Q]	[76.6-85.4]	[17,526.3-26,337.8]	[56.6-58.8]	[100.0-100.0]	[99.9-100.0]	[99.6-99.9]	[98.7-99.7]	[80.0-96.5]
Benign (n = 10)	Median	85.2	29,686.5	60.9	100.0	99.9	99.9	99.1	76.9
	[1Q-3Q]	[83.2-89.0]	[20,312.5-34,080.3]	[56.7-62.4]	[100.0-100.0]	[99.8-100.0]	[99.7-99.9]	[98.6-99.7]	[70.9-93.3]

Supplementary Table S3. Information of patients with metastatic ccRCC

ID	Sex	Age	Tumor size (cm)	pT	Grade	ctDNA variant		VAF(%)
KC_062	M	52	6.8	3a	3	*MTOR*	C1483R	15.627
						VHL	L169P	9.489
						PBRM1	F1100fs	6.660
						TP53	G245C	6.268
KC_093	F	71	7.0	3a	3	*NF1*	S1497fs	0.263
KC_104	M	60	9.0	3a	4	*PTEN*	R130X	6.661
						PBRM1	P1272fs	5.235
						VHL	Splicing	4.437
						BAP1	R114H	0.257
						NF1	D1849E	0.104
KC_088	M	81	11.0	3a	3	*TP53*	C238Y	1.126
						KDM6A	L361F	0.737
KC_032	M	52	12.5	3a	3	*PBRM1*	R522Q	1.573
						PTEN	K128fs	1.526
						VHL	R69fs	1.21
KC_019	M	69	17.0	3a	4	*SETD2*	L1923fs	0.907
						VHL	L128P	0.733
						PTEN	R173P	0.595
						PBRM1	K907fs	0.370
KC_087	F	69	4.3	3a	3	Not detected	-	-
KC_065	M	64	12.0	4	3	Not detected	-	-

Abbreviations: F, female; M, male; pT, pathological T stage; VAF, variant allele frequency

Supplementary Table S4. Information of patients with pT1b-3a ccRCC

ID	Sex	Age	Tumor size (cm)	pT	Grade	ctDNA variant		VAF(%)
KC_020	F	64	5.4	1b	1	*TP53*	Y220C	0.442
						SETD2	P2361H	0.422
KC_036	M	79	4.1	1b	2	*EGFR*	A647T	0.141
KC_119	F	51	6.5	1b	2	Not detected	-	-
KC_060	M	63	6.4	1b	2	Not detected	-	-
KC_079	M	41	5.5	1b	2	Not detected	-	-
KC_048	M	79	8.0	2a	3	*TP53*	P153fs	0.178
KC_078	M	48	9.0	2a	3	Not detected	-	-
KC_086	F	44	8.5	2a	3	Not detected	-	-
KC_058	M	86	9.5	3a	4	*EGFR*	R958C	0.191
KC_015	M	63	6.9	3a	3	*PBRM1*	Y1506H	0.489
KC_094	M	59	5.5	3a	3	*NF1*	P2221L	0.167
KC_113	M	42	6.8	3a	2	Not detected	-	-
KC_031	M	64	6.5	3a	3	Not detected	-	-
KC_061	M	32	2.2	3a	2	Not detected	-	-

Abbreviations: F, female; M, male; pT, pathological T stage; VAF, variant allele frequency

Supplementary Table S5. Information of patients with benign renal tumor

ID	Sex	Age	Tumor size (cm)	Type	ctDNA variant		VAF(%)
KC-089	F	66	0.7	Angiomyolipoma	Not detected	-	-
KC-092	M	42	0.7	Angiomyolipoma	Not detected	-	-
KC-077	F	47	0.8	Angiomyolipoma	Not detected	-	-
KC-021	F	62	1.1	Angiomyolipoma	Not detected	-	-
KC-063	F	38	1.5	Simple cortical cyst	Not detected	-	-
KC-035	F	45	1.6	Angiomyolipoma	Not detected	-	-
KC-033	F	57	2.0	Oncocytoma	Not detected	-	-
KC-027	M	61	3.3	Oncocytoma	Not detected	-	-
KC-017	F	59	6.5	Angiomyolipoma	Not detected	-	-
KC-103	M	58	8.0	Pseudocyst	*MET*	R426H	0.02859

Abbreviations: F, female; M, male; VAF, variant allele frequency

Supplementary Table S6. List of ctDNA variants of patients with pT1a ccRCC

ID	Sex	Age	Tumor size (cm)	Grade	ctDNA variant		VAF(%)
KC_098	M	56	1.2	1	*PIK3CA*	Q1033L	0.115
KC_101	F	71	1.3	2	*KDM6A*	Q367X	0.216
KC_057	M	56	1.7	3	*TP53*	R196Q	0.453
KC_004	F	50	1.9	2	*PTEN*	Y16X	0.499
KC_059	M	59	2.2	2	*NF1*	K513X	0.350
KC_007	M	63	2.5	2	*PTEN*	Splicing	2.749
KC_118	F	51	2.8	3	*MET*	R987Q	0.378
KC_083	F	62	3	3	*KDM6A*	E1335fs	0.614
KC_069	M	68	3.8	1	*EGFR*	Splicing	0.477
KC_121	M	62	3.8	4	*VHL*	M1I	0.283
KC_025	M	47	4	2	*PIK3CA*	S235F	0.232

Abbreviations: F, female; M, male; pT, pathological T stage; VAF, variant allele frequency
